# Optimizing Immunization Strategies for the Induction of Antigen-Specific CD4 and CD8 T Cell Responses for Protection against Intracellular Parasites

**DOI:** 10.1128/CVI.00251-16

**Published:** 2016-09-06

**Authors:** Kimberly A. Hofmeyer, Malcolm S. Duthie, John D. Laurance, Michelle A. Favila, Neal Van Hoeven, Rhea N. Coler, Steven G. Reed

**Affiliations:** Infectious Disease Research Institute, Seattle, Washington, USA; Food and Drug Administration

## Abstract

Immunization strategies that generate either CD4 or CD8 T cell responses are relatively well described, but less is known with regard to optimizing regimens to induce both CD4 and CD8 memory T cells. Considering the importance of both CD4 and CD8 T cells in the control of intracellular pathogens such as Leishmania donovani, we wanted to identify vaccines that could raise both CD4 and CD8 T cell responses and determine how to configure immunization strategies to generate the best combined protective T cell response. We examined responses generated against the Leishmania vaccine antigen F3 following its administration in either recombinant form with the Toll-like receptor 4 (TLR4) agonist-containing adjuvant formulation GLA-SE (F3+GLA-SE) or as a gene product delivered in an adenoviral vector (Ad5-F3). Homologous immunization strategies using only F3+GLA-SE or Ad5-F3 preferentially generated either CD4 or CD8 T cells, respectively. In contrast, heterologous strategies generated both antigen-specific CD4 and CD8 T cells. Administration of F3+GLA-SE before Ad5-F3 generated the greatest combined CD4 and CD8 responses. Cytotoxic CD8 T cell responses were highest when Th1 cells were generated prior to their induction by Ad5-F3. Finally, a single immunization with a combination of F3+GLA-SE mixed with Ad5-F3 was found to be sufficient to provide protection against experimental L. donovani infection. Taken together, our data delineate immunization regimens that induce antigen-specific CD4 and CD8 T cell memory responses, and identify a single immunization strategy that could be used to rapidly provide protection against intracellular pathogens in regions where access to health care is limited or sporadic.

## INTRODUCTION

Although vaccines that promote T cell responses are relatively limited in the clinical setting, a plethora of preclinical vaccine studies have described the generation of CD4 and CD8 T cells. Initially these studies exclusively partitioned the vaccines for the generation of either CD4 or CD8 T cells, however, and interplay between these cell types was generally overlooked. More recent studies, predominantly of viral infections for which the principal immune protection is provided by CD8 T cells, have indicated the important contribution of CD4 T cell help in generating and maintaining effective CD8 T cells (1).

Leishmania donovani is an important human pathogen that is transmitted during the blood meal of infected sand flies. This can lead to disseminated infection that manifests as visceral leishmaniasis (VL). Because it is generally fatal if left untreated, VL causes an estimated 20,000 to 40,000 deaths per year (2, 3). Interestingly, despite the high incidence of disease, it is estimated that up to 90% of L. donovani infections in humans remain subclinical and do not cause symptoms (4). Asymptomatic infections that resolve without manifesting VL are believed to be controlled when an effective antigen-specific cell-mediated immune response is generated. T cells of asymptomatic L. donovani-infected individuals, and also patients cured of VL, respond to Leishmania antigen by producing gamma interferon (IFN-γ) (5–7). In contrast, active VL disease is associated with a depressed response and infected macrophages are rendered unresponsive to activating cytokines by the presence of interleukin 10 (IL-10) (5, 8–12). These data indicate the importance of a potent Th1 response, rather than a Th2 or suppressive microenvironment, to protection (13). In addition, CD8 T cells from healed VL patients produce granzyme B, in contrast to the CD8 T cells of patients with chronic VL, which have an exhausted phenotype (5, 14). Thus, reports have indicated the critical importance of either CD4 or CD8 T cells in control of L. donovani infection. The ideal vaccine for the prevention of L. donovani infection and VL would appear to be one that generates both memory CD4 T cells capable of producing Th1 cytokines and cytotoxic CD8 T cells that, together, can help in the killing of intracellular parasites to prevent progression to disease.

In addition to its own clinical importance, as an experimental system L. donovani infection also lends itself to understanding how to generate both CD4 and CD8 T cells that are antigen specific and functionally protective. Several vaccines that elicit CD4 or CD8 T cell responses have demonstrated protection in mouse models of VL, but the specific contribution of each cell type is generally unclear (15–17). We recently reported that a chimeric protein expressed from a fusion of two Leishmania genes, named F3, when formulated with GLA-SE, a synthetic Toll-like receptor 4 (TLR4) agonist in an oil-in-water stable emulsion, generated F3-specific, CD4 T cell-dependent protection in a mouse model of L. donovani infection (18, 19). Defined subunit vaccines involving recombinant proteins typically generate robust antigen-specific Th1 responses but do not appear to induce CD8 T cells (18, 20, 21).

Considering the importance of both CD4 and CD8 T cells in the control of L. donovani infection, we wanted to identify vaccines that could raise both CD4 and CD8 T cell responses and determine how to configure immunization strategies using these vaccines to generate the best combined protective T cell response. We therefore developed an adenoviral vector expressing the F3 protein (Ad5-F3), to contrast with and potentially complement the F3+GLA-SE vaccine. To determine the strategy that induced the optimal combination of antigen-specific CD4 and CD8 T cell responses, capable of protecting against L. donovani infection, we evaluated various prime-boost regimens with these vaccines. We also developed a strategy to invoke these responses as quickly and practically as possible, discovering that a single simultaneous immunization with Ad5-F3 and F3+GLA-SE was sufficient to provide protection.

## MATERIALS AND METHODS

### Mice and immunizations.

Female C57BL/6 mice (purchased from Charles River Laboratories, Wilmington, MA) were maintained under specific-pathogen-free conditions and in accordance with animal procedures approved by the IDRI institutional animal care and use committee. Mice entered experiments at 6 to 8 weeks of age and were immunized by subcutaneous injection of vaccines at the base of the tail. The recombinant antigen-containing vaccines (F3+GLA-SE, F3+SE, and F3 only) were prepared to provide a total of 5 μg/dose of protein, GLA was used at 5 μg/dose, and each vaccine was delivered in a total volume of 0.1 ml. The Ad5-F3 vaccine was prepared to provide a total of 1 × 10^7^ PFU/dose in a total volume of 0.1 ml. Homologous and heterologous prime-boost regimens with these two vaccines involved a priming event followed 6 weeks later with a booster immunization.

### Antigen-specific antibody responses.

Blood was collected, serum was prepared, and antigen-specific antibody responses were analyzed by enzyme linked immunosorbent assay (ELISA) for IgG2 and IgG1 isotypes. Briefly, ELISA plates (Nunc, Rochester, NY) were coated with 1 μg/ml of antigen in 0.1 M bicarbonate buffer and blocked with 0.1% bovine serum albumin–phosphate-buffered saline (BSA-PBS). Then, in consecutive order and following washes in PBS-Tween, serially diluted serum samples, anti-mouse IgG1-horseradish peroxidase (IgG1-HRP) or anti-mouse IgG2a-HRP (Southern Biotech, Birmingham, AL), and 2,2′-azinobis(3-ethylbenzthiazolinesulfonic acid) (ABTS)-H_2_O_2_ (Kirkegaard and Perry Laboratories, Gaithersburg, MD) were added to the plates. Plates were analyzed at 405 nm (EL_X_808; Bio-Tek Instruments Inc., Winooski, VT). Endpoint titer was determined as the last optical density (OD) value greater than a threshold determined by sera from saline-immunized control mice.

### Antigen-specific T cell responses.

Spleens were removed 4 or 16 weeks after the final immunization and single-cell suspensions prepared. Mononuclear cells were enumerated using a ViaCount assay with a PCA system (Guava Technologies, Hayward, CA). Cells were cultured at 1 × 10^6^ cells per well in duplicate in a 96-well plate (Corning Incorporated, Corning, NY) in RPMI 1640 supplemented with 5% heat-inactivated fetal calf serum (FCS) and 50,000 U of penicillin-streptomycin (Invitrogen). The F3-specific CD4 T memory response was determined following incubation of spleen cells with 10 μg/ml of F3 antigen. The F3-specific CD8 T memory response was determined following incubation of spleen cells with a pool of two major F3 major histocompatibility complex class I (MHC-I) peptides. MHC-I peptides (H-2b) were predicted using the SYFPEITHI database (22) and confirmed by the presence of IFN-γ-producing CD8 T cells following recall of splenocytes from C57BL/6 mice immunized with Ad5-F3 (data not shown). Boolean gate analysis was performed to determine the proportion of CD44hi CD4 and CD8 T cells producing combinations of CD154, IFN-γ, tumor necrosis factor (TNF), or IL-2; cells were characterized by production of all four, any combination of two or three, or any single molecule. Degranulation and cytotoxic capability of CD8 T cells was indicated by detecting either granzyme B or the surrogate molecule CD107a, a lysosome-associated protein that is exposed to the cell surface during degranulation (23). All antibodies were purchased from eBioscience, Inc., San Diego, CA.

### CD8 T cell cytotoxicity.

To determine the cytotoxicity of CD8 T cells, we conducted an *in vivo* target cell killing assay (24). Spleen cells were incubated with the pool of two major F3 MHC-I peptides or medium only and labeled with high or low concentrations of carboxyfluorescein succinimidyl ester (CFSE; eBioscience). CFSE-labeled spleen cells were mixed 1:1 and intravenously injected into recipient mice. After 18 h, spleens were removed from recipient mice and the proportions of transferred cells determined by flow cytometry. Specific killing was quantified by determining the relative percentage of F3 peptide-pulsed (CFSE^hi^) cells lost in immunized mice compared to that in saline-immunized control mice.

### Infection and determination of parasite burden.

L. donovani (MHOM/SD/00/1S-2D) parasites were passed through Syrian golden hamsters to generate virulent amastigote and promastigote stocks in M199 medium. Infection was initiated by injection of 1 × 10^6^ stationary-phase L. donovani organisms (MHOM/SD/00/1S-2D) into the vein in the eye socket. One month after inoculation, livers were harvested and homogenized and parasite burden calculated by real-time PCR. DNA was extracted from homogenate using QIAmp DNA minikits (Qiagen) and quantified using a NanoDrop UV-Vis spectrophotometer (ND-1000). L. donovani DNA was detected using primers for the Leishmania infantum DNA repeat region (GenBank accession no. L42486 (forward, 5′-GCGACGTCCGTGGAAAGAA-3′, and reverse, 5′-GGCGGGTACACATTAGCAGAA-3′) with a 6-carboxyfluorescein (FAM) reporter sequence (5′-CAACGCGTATTCCC-3′) that detects a 203-bp genomic repeat region specific to Leishmania species (NCBI BLASTN). Mouse glyceraldehyde-3-phosphate dehydrogenase (GAPDH) FAM (Life Technologies) was used as an internal reference control. The number of parasite per microliter of DNA was determined by extrapolating the quantification cycle (*C_q_*) of each sample against a standard curve generated with known quantities of parasites, and then burdens were expressed as number of parasites per liver.

### Statistical analyses.

Statistical analyses were conducted using one-way analysis of variance and Dunnett's or Tukey's multiple-comparison test used to compare two groups. Statistical significance was considered when the *P* values were <0.05.

## RESULTS

### Differential generation of CD4 T cell memory by F3-containing vaccines.

**FIG 1:**
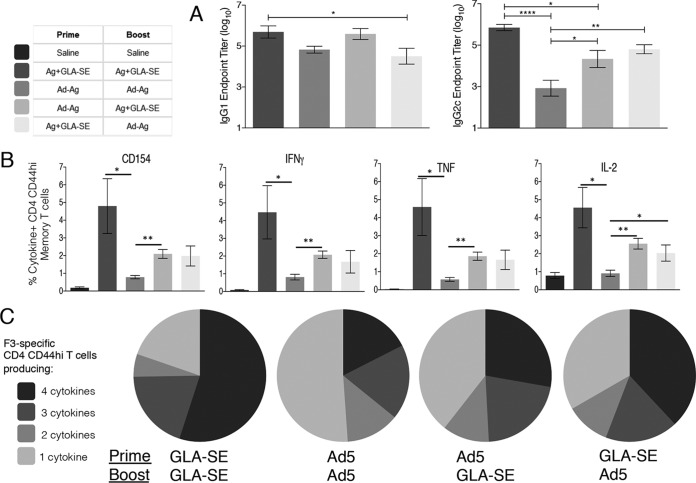
Immunization regimens that include a GLA-SE-formulated protein vaccine generate memory CD4 T cells. C57BL/6 mice were primed, and 6 weeks later boosted, with the indicated vaccines. Sixteen weeks after the boost, blood was collected and F3-specific serum antibodies were determined (A). In addition, spleens were removed and single-cell suspensions prepared. Cells were incubated with F3 protein and analyzed by flow cytometry for expression of CD154 or the indicated cytokine (B). Data are means and SEM (*n* = 5 per group). (C) Relative proportions of CD4 T cells expressing combinations of each cytokine/CD154. Data are representative of results obtained in 2 or 3 independent experiments. *, **, and ****, *P* < 0.05, *P* < 0.01, and *P* < 0.0001, respectively, for the indicated groups. In panel B, with the exception of Ad-Ag/Ad-Ag-induced IL-2-producing CD4 CD44^hi^ cells, all immunization-induced responses were significantly greater (*P* < 0.05) than those observed following saline/saline injection.

Vaccines involving recombinant antigens formulated with an appropriate adjuvant are typically good at inducing Th1 cells. Our previous work indeed demonstrated that a series of three F3+GLA-SE immunizations generates a robust antigen-specific CD4 T cell memory response (18). As a preliminary indicator of immune biasing, we assessed the antigen-specific antibody responses of mice immunized with either the F3+GLA-SE alone, an adenoviral vector with an F3-encoding insert alone (Ad5-F3 vaccine), or heterologous iterations of these vaccines in a prime and single-boost strategy. Antigen-specific IgG1 responses in all groups were comparable (Fig. 1A). In contrast, two immunizations with F3+GLA-SE promoted antigen-specific IgG2c responses that were slightly higher than those of mice that received single immunizations with F3+GLA-SE as part of the heterologous strategies (Fig. 1A). The anti-F3 IgG2c responses in mice that received only the Ad5-F3 vaccine was significantly lower (Fig. 1A). These data suggest generation of Th1 responses in mice immunized with recombinant protein but poor induction in mice immunized with adenovirus-delivered antigen.

To evaluate antigen-specific CD4 T cell responses directly, memory CD44^hi^ CD4 T cells were identified among spleen cells from immunized mice following incubation with the F3 antigen. Activated antigen-specific cells, as indicated by CD154 (CD40L) expression, were induced by all of the immunization regimens (Fig. 1B; all *P* values <0.05 versus the value for the saline group). Homologous prime-boost with F3+GLA-SE generated the most robust CD4 T cell memory response as revealed by cells producing IFN-γ, TNF, or IL-2 (Fig. 1B and C). Irrespective of whether F3+GLA-SE was used in the prime or in the boost, the heterologous prime-boost strategies generated lower, but still readily detectable, numbers of F3-specific CD4 T cells that also produced these Th1 cytokines. The homologous prime-boost strategy with Ad5-F3 generated only small populations of F3-specific CD4 T cells, and these populations were significantly reduced relative to their comparative populations induced by homologous F3+GLA-SE immunization (Fig. 1B). Given that the proportion of polyfunctional antigen-specific CD4 T cells, revealed by the simultaneous production of IFN-γ, TNF, or IL-2 on an individual-cell basis, has been demonstrated to positively correlate with vaccine-mediated protection against pathogens, including Leishmania (25), we also determined the proportion of CD4^+^ CD44^hi^ T cells producing combinations of CD154, IFN-γ, TNF, or IL-2. Among the vaccine strategies evaluated, the homologous prime-boost regimen with F3+GLA-SE promoted the highest proportion of memory CD4 T cells producing two or more cytokines, while the homologous regimen with Ad5-F3 had the lowest (Fig. 1C). Irrespective of whether F3+GLA-SE was the prime or the boost, the two heterologous regimens generated similar compositions of antigen-specific CD4 T cells (Fig. 1C). Taken together, these data demonstrate the importance of recombinant antigen in directing the CD4 T cell response.

### Differential generation of CD8 T cell memory by F3-containing vaccines.

**FIG 2:**
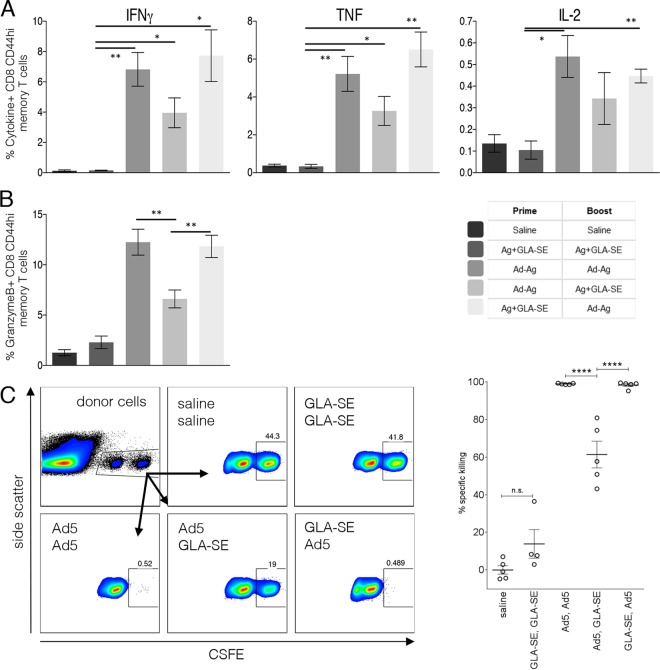
Immunization regimens that include an Ad5-based vaccine generate memory CD8 T cells. C57BL/6 mice were primed, and 6 weeks later boosted, with the indicated vaccines. Sixteen weeks after the boost, spleens were removed and single-cell suspensions prepared. Cells were incubated with F3 MHC-I peptides and analyzed by flow cytometry for expression of the indicated cytokine (A) or granzyme B (B). To determine killing by CD8 memory T cells, control and peptide-pulsed spleen cells from untreated mice were stained with a low (0.2 μM) or high (2 μM) concentration of CFSE and mixed at a 1:1 ratio (donor cells) (C). Donor cells were injected intravenously into immunized mice and, 18 h later, recovered from the spleens. Data are representative flow cytometry plots, with the previous immunization regimen indicated, and specific killing as determined by contrasting the ratio of control and peptide-pulsed cells recovered. Data are means and SEM (*n* = 5 per group) and are representative of results obtained in 2 or 3 independent experiments. * and **, *P* < 0.05 and *P* < 0.01, respectively, for the indicated groups. In panels A and B, with the exception of Ag+GLA-SE/Ag+GLA-SE, immunization-induced responses were significantly greater (*P* < 0.05) than those observed following saline/saline injection. n.s., not significant.

We next assessed antigen-specific CD8 T cell responses. As expected, antigen-specific CD8 T cells could not be detected after immunization with F3+GLA-SE alone (Fig. 2A; not significantly different from the value for saline-treated mice). This contrasted with the Ad5-F3-containing regimens, which all generated F3-specific CD44^hi^ CD8^+^ memory T cells that produced IFN-γ, TNF, or IL-2 upon incubation with antigen (Fig. 2A; all *P* values < 0.05 versus the value for the saline group). As suggested by the high proportions of antigen-specific CD8 T cells that expressed granzyme B, a strong CD8 T cell cytotoxic capacity was also generated by the homologous Ad5-F3 regimen (Fig. 2B). Similar proportions of granzyme B-expressing CD8 T cells were observed in mice immunized with the F3+GLA-SE prime/Ad5-F3 boost regimen, whereas the Ad5-F3 prime/F3+GLA-SE boost regimen generated a smaller granzyme B-expressing CD8 T cell population (Fig. 2B). Specific killing of MHC class I-restricted F3-peptide-pulsed cells mirrored the proportions of granzyme B-expressing CD8 T cells, such that between the Ad5-F3 containing regimens, the Ad5-F3 prime and subsequent F3+GLA-SE boost generated the lowest proportion of antigen-specific CD8 T cells (Fig. 2C). In contrast, an Ad5-F3 boost after antigen-specific CD4 (by F3+GLA-SE) or CD8 (by Ad5-F3) T cells had already been generated led to significantly larger proportions of cytotoxic CD8 T cells. These data emphasize the importance of intracellular delivery of antigen, in this case via an adenoviral vector, in generating the CD8 T cell response.

### Th1 cells facilitate the expansion of antigen-specific CD8 T cells.

**FIG 3:**
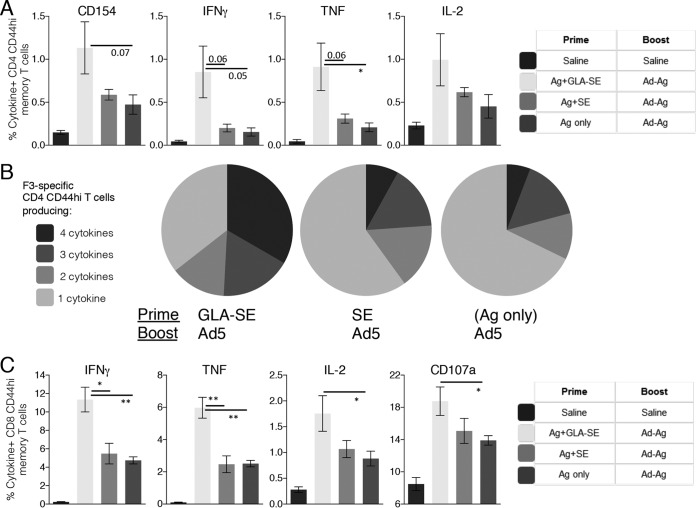
Quality of preexisting F3-specific CD4 T cells impacts the Ad5-F3 boosting effect. C57BL/6 mice were primed as indicated to generate different qualities of F3-specific CD4 T cells and 6 weeks later boosted by injection of Ad5-F3. Sixteen weeks after the boost, spleens were removed and single-cell suspensions prepared. Cells were incubated with the F3 protein and analyzed by flow cytometry for expression of CD154 or the indicated cytokine (A). Data are means and SEM (*n* = 5 per group). (B) Relative proportions of CD4 T cells expressing combinations of each cytokineCD154. (C) Cells were incubated with F3 MHC-I peptides and analyzed by flow cytometry for expression of CD107a or the indicated cytokine. Data are means and SEM (*n* = 5 per group) and are representative of results obtained in 2 or 3 independent experiments. * and **, *P* < 0.05 and *P* < 0.01, respectively, for the indicated groups. The actual *P* value is indicated in comparisons where values of <0.1 were assessed.

To determine if the presence, or quality, of a preexisting F3-specific CD4 T cell response impacted the ability of Ad5-F3 to generate responses, we immunized mice with different CD4 T cell-biasing vaccine formulations (18, 21, 26). Mice were primed with F3+GLA-SE to generate antigen-specific Th1 cells, with F3+SE to generate antigen-specific Th2 cells, or with the F3 protein alone to generate only a small population of antigen-specific Th cells. Mice primed with F3+SE or F3 alone and then boosted with Ad5-F3 generated fewer F3-specific CD4 T cells than the F3+GLA-SE-primed mice (Fig. 3A). In addition, the CD4 T cell populations from these mice also had lower proportions of Th1 cytokine-producing cells than those observed in mice that were primed with F3+GLA-SE (Fig. 3A and B). With regard to CD8 T cells, priming with F3+GLA-SE prior to the Ad5-F3 immunization resulted in more antigen-specific memory CD8 T cells producing IFN-γ, TNF, or IL-2 than did priming with either F3+SE or F3 alone (Fig. 3C). Similarly, CD8 T cells from mice primed with F3+GLA-SE before the Ad5-F3 boost exhibited greater degranulation in response to TCR engagement than CD8 T cells from mice primed with either F3+SE or F3 only (Fig. 3C, CD107a).

**FIG 4:**
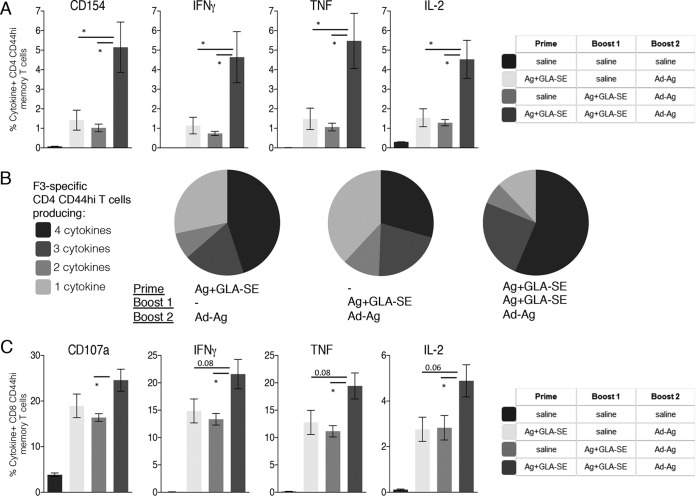
Magnitude of the preexisting F3-specific Th1 cell population impacts the Ad5-F3 boosting effect. C57BL/6 mice were immunized as indicated to generate different magnitudes of the F3-specific CD4 T cell population and 6 weeks later boosted by injection of Ad5-F3. Sixteen weeks after the boost, spleens were removed and single-cell suspensions prepared. Cells were incubated with the F3 protein and analyzed by flow cytometry for expression of CD154 or the indicated cytokine (A). Data are means and SEM (*n* = 5 per group). (B) Relative proportion of CD4 T cells expressing combinations of each cytokine/CD154. (C) Cells were incubated with F3 MHC-I peptides and analyzed by flow cytometry for expression of CD107a or the indicated cytokine. Data are means and SEM (*n* = 5 per group) and are representative of results obtained in 2 or 3 independent experiments. *, *P* < 0.05 for the indicated groups. The actual *P* value is indicated in comparisons where values of <0.1 were assessed. In panels A and C, all immunization induced responses were significantly greater (*P* < 0.05) than those observed following saline-only injections.

Mice were also immunized with F3+GLA-SE for a second time prior to the Ad5-F3 immunization to evaluate if an increased population of CD4 T cells further enhanced the boost by Ad5-F3. The F3-specific CD4 T cell response was indeed increased in magnitude with the additional immunizations (data not shown). Compared with responses of mice that received a single F3+GLA-SE immunization before an Ad5-F3 boost, generating a larger pool of CD4 T cells by immunizing twice with F3+GLA-SE significantly enhanced the Ad5-F3 boosting of CD4 and CD8 T cell memory responses (Fig. 4). Taken together, these data indicate an important contribution of antigen-specific Th1 cells in the subsequent generation of cytotoxic CD8 T cells.

### Simultaneous immunization with F3+GLA-SE and Ad5-F3 is immunogenic.

**FIG 5:**
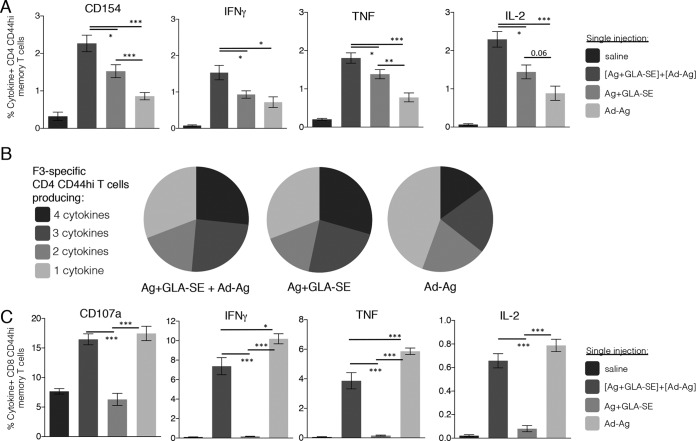
Generation of F3-specific CD4 and CD8 T cells by a single injection of vaccine(s). C57BL/6 mice received a single immunization with the indicated vaccines. Four weeks after the boost, spleens were removed and single-cell suspensions prepared. Cells were incubated with the F3 protein and analyzed by flow cytometry for expression of CD154 or the indicated cytokines (A). Data are means and SEM (*n* = 5 per group). (B) Relative proportions of CD4 T cells expressing combinations of each cytokine/CD154. (C) Cells were incubated with F3 MHC-I peptides and analyzed by flow cytometry for expression of CD107a or the indicated cytokines. Data are means and SEM (*n* = 5 per group) and are representative of results obtained in 2 or 3 independent experiments. *, **, and ***, *P* < 0.05, *P* < 0.01, and *P* < 0.001, respectively, for the indicated groups. The actual *P* value is indicated in comparisons where values of <0.1 were assessed.

Having identified that preexisting CD4 T cells influence the anti-F3 CD8 T cell response, we then examined if these responses could be generated concurrently. We evaluated the responses of mice that were immunized with the F3+GLA-SE and Ad5-F3 vaccines combined and provided in a single injection. Mice that received a single immunization with the mixed vaccine generated significantly more F3-specific Th1 cells, as well as higher proportions of IFN-γ-, TNF-, or IL-2-producing Th1 cells, than mice immunized once with either F3+GLA-SE or Ad5-F3 alone (Fig. 5A and B). Although the mixed vaccine generated slightly lower proportions of cytokine-producing CD8 T cells than were elicited by a single immunization with Ad5-F3 alone, these immunizations demonstrated similar abilities to generate cytotoxic CD8 T cells (Fig. 5C, CD107a). Thus, a single immunization with a mixture of F3+GLA-SE and Ad5-F3 generated both antigen-specific CD4 and CD8 T cells without any appreciable reduction in immunogenicity relative to provision of these vaccines alone.

### Immunization schemes that protect against experimental L. donovani infection.

**FIG 6:**
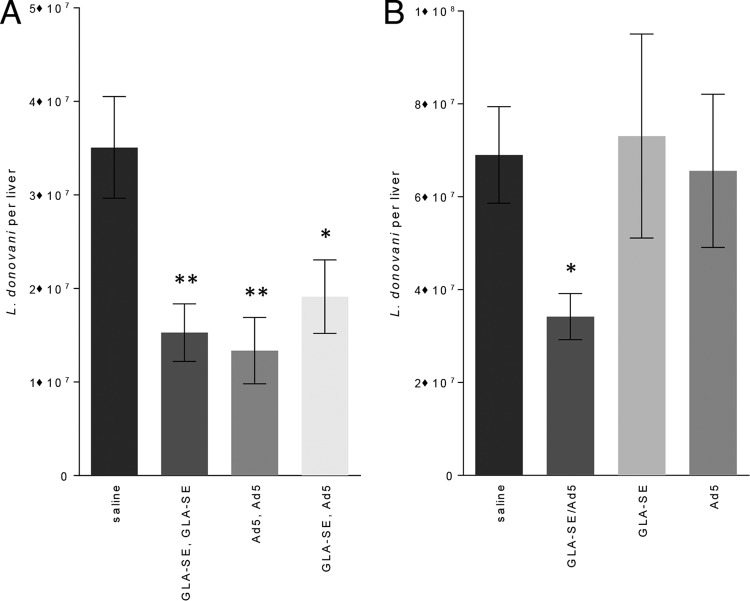
Protection against L. donovani infection by a single injection. C57BL/6 mice were immunized with the indicated homologous or heterologous regimen (A) or a single injection (B) of vaccines. Mice were infected by intravenous inoculation of L. donovani parasites 4 weeks after their final, or only, immunization. Four weeks after infection, livers were removed and parasite burden was determined for each animal by PCR. Data are representative of results obtained in 2 independent experiments and are means and SEM (*n* = 7 per group). * and **, *P* < 0.05 and *P* < 0.01, respectively, compared with the value for the unvaccinated group.

Having demonstrated that homologous and heterologous strategies using F3+GLA-SE and Ad5-F3 raised either antigen-specific CD4 or CD8 T cells, or both, we assessed if these responses were biologically meaningful and could protect against L. donovani infection. Homologous immunizations with either F3+GLA-SE or Ad5-F3 significantly reduced liver parasite burdens in L. donovani-infected mice (Fig. 6A). Parasite numbers were also significantly reduced in mice that were immunized with the heterologous F3+GLA-SE prime/Ad5-F3 boost regimen (Fig. 6A). From a practical standpoint, immunization with antigen in these distinct vaccine platforms would be easier to implement if, rather than in a heterologous F3+GLA-SE prime/Ad5-F3 boost regimen requiring injections on different days, the vaccines could be provided at the same time. Considering the immune responses generated by a single immunization with the mixture of these vaccines, we were not surprised to find that relative to those in unimmunized mice, parasite burdens were also reduced in mice immunized with F3+GLA-SE mixed with Ad5-F3 (Fig. 6B). In stark contrast, a single immunization with either F3+GLA-SE or Ad5-F3 alone did not confer protection. Thus, either two immunizations, or even a single injection, with a mixture of the F3+GLA-SE and Ad5-F3 vaccines are potent enough to protect against L. donovani infection.

## DISCUSSION

While preclinical reports of vaccines with the intent of inducing T cell responses are abundant, these are typically partitioned for the generation of either CD4 or CD8 T cells. In the clinical setting T cell vaccines are much more limited, but generation of both CD4 and CD8 T cells is, if not critical, at least very appealing. In this study, we confirmed that homologous prime-boost immunizations with the defined subunit vaccine F3+GLA-SE elicited a robust antigen-specific CD4 T cell response but not detectable CD8 T cell responses. Conversely, homologous prime-boost immunizations with a viral vector-based vaccine, Ad5-F3, generated a robust CD8 T cell response but only a limited CD4 T cell response. These immunization schemes provide the opportunity to investigate the interplay of CD4 and CD8 T cells in a vaccine setting and in an infectious setting for which protective roles have been attributed to both cell types. Our data clearly demonstrate that while the order of the F3-containing vaccines within the heterologous regimens did not have a significant impact on the magnitude or quality of the antigen-specific CD4 T cell response, there was a clear benefit in using F3/GLA-SE before Ad5-F3 for the antigen-specific CD8 T cell response. Furthermore, generation of antigen-specific Th1 cells, but less so Th2 cells, is beneficial for the subsequent generation of cytotoxic CD8 T cells. Overall, an F3+GLA-SE prime/Ad5-F3 boost strategy induced the best combined CD4 and CD8 T cell memory response. For practical administration, our data also importantly demonstrate that a single immunization with a mixture of these vaccines can generate a protective immune response against an intracellular pathogen.

Various lines of investigation demonstrate that CD4 T cells can strongly influence CD8 T cell responses: the absence or removal of CD4 T cells during a primary immune response results in a defective CD8 T cell response (27–30), preexisting antigen-specific CD4 T cells can enhance primary CD8 T cell responses (27, 31), and strategies employing a “protein first” immunization prior to a CD8 T cell-priming immunization facilitate a robust CD8 T cell response (20, 32–34). We therefore hypothesized that F3-specific CD4 T cells would influence any subsequent F3-specific CD8 T cell response. Our data indicate that generating a weak (by F3 only) or Th2-biased (by F3+SE) CD4 T cell response did not support the generation of antigen-specific CD8 T cells by subsequent Ad5-F3 immunization. Immunization with F3+GLA-SE prior to Ad5-F3 administration did, however, generate a strong CD8 T cell response. These responses were at levels greater than the CD8 T cell response generated by the reverse immunization regimen, i.e., generating CD8 T cells before CD4 T cells. This suggests that existing memory Th1 cells generated by F3-GLA-SE supported CD8 T cell priming to generate a more robust response. Boosting the Th1 response with a second F3+GLA-SE immunization further aided generation of the CD8 T cell response by Ad5-F3. It is still somewhat unclear whether the critical aspect of the CD4 T cell response generated by F3+GLA-SE to support the CD8 T cell response is the magnitude of the CD4 T cell population, the quality of the Th1 cells generated, or the relative combination. Regardless, our data demonstrate the benefit of, and importance of generating, a Th1 response in optimizing vaccine strategies that have a goal of generating cytotoxic CD8 T cells.

Patient studies suggest that CD8 T cell cytotoxicity is at least partially protective against other intracellular pathogens (35, 36), and while CD8 T cells from cured VL patients produce granzyme B (14), a clear role for CD8 T cells in human VL has not yet been demonstrated. Macrophage-activating cytokines, such as IFN-γ and TNF, are critical to the control of Leishmania infection and progression to active VL and are produced by both Th1 and CD8 T cells (6, 7, 10, 14). We previously found the F3+GLA-SE vaccine to be protective in a mouse model of VL and, based on cell depletion studies, that protection was dependent on CD4 T cells; the vaccine did not generate a CD8 T cell response (18). Other vaccines tested in mouse models of VL that induced protection generated both a CD4 and CD8 T cell response; however, cell depletion studies were not conducted and protection could not be ascribed to any particular cell type (15–17). Given that roles for both CD4 and CD8 T cells in the control of Leishmania infection have been described, a prudent and practical vaccine strategy may involve generation of both T cell types. L. donovani parasites in which centrin has been deleted (*LdCen1*^−/−^) have a dramatically attenuated phenotype and are cleared within 12 weeks of inoculation into mice (37). Mice inoculated with *LdCen1*^−/−^ parasites developed Leishmania-specific CD4 and CD8 T cells expressing Th1 cytokines and were protected against virulent L. donovani challenge (37). Trials of *LdCen1*^−/−^ parasites as a vaccine have been conducted with dogs, and reduced parasite numbers are reported for immunized animals following intravenous L. infantum challenge (38, 39). We found that a single immunization involving a mixture of the defined F3+GLA-SE and Ad5-F3 vaccines not only was immunogenic but also was protective against L. donovani infection. This contrasted with single immunizations with F3+GLA-SE or Ad5-F3 vaccines alone, which, despite generating CD4 or CD8 T cell responses, did not provide protection. Although it is unclear which T cell type is critical to the protection conferred by the F3+GLA-SE/Ad5-F3 vaccine, the likelihood is that both T cell subsets are involved. The F3+GLA-/Ad5-F3 vaccine generated a CD8 T cell response equivalent to that generated by the Ad5-F3 vaccine and a CD4 T cell response that was more robust than with either vaccine alone.

In studies similar to ours, a combination vaccine consisting of protein plus viral vector plus GLA-AF used as a booster immunization significantly augmented the T cell response induced by an HIV vaccine in mice and rabbits (40). Coadministration of a lentiviral vector vaccine with GLA-AF induced robust CD4 and CD8 T cells that reduced tumor growth and mortality in a mouse cancer model (41). This suggests that the GLA-SE and Ad5-F3 components of the mixed vaccine, but not necessarily the recombinant protein, are the important components for the immunogenicity and protection observed in our current studies. Determining which individual components of the mixed vaccine (F3 antigen, GLA-SE, or Ad5-F3) critically contribute to immunogenicity and protection could allow further refinement and simplification of the vaccine formulation.

Our data identify immunization regimens that induce antigen-specific memory T cell responses, involving both CD4 and CD8 T cells, that protect against infection with L. donovani. In many regions where visceral leishmaniasis is endemic, consistent and regular access to health care is limited. The ability to induce protective immunity with a single immunization, such as we observed with a mixture of F3/GLA-SE and Ad5-F3, appears to be a good and practical control option that would be highly beneficial in such regions. This property is also extremely attractive where an established vaccine platform/regimen generating mixed CD4 and CD8 T cells could be rapidly adapted and deployed to serve as a component of a public health emergency response to control an emerging infectious disease outbreak.
